# The Year in Pediatric Electrophysiology: 2021

**DOI:** 10.19102/icrm.2022.130111

**Published:** 2022-01-15

**Authors:** Johannes C. von Alvensleben, Kathryn K. Collins

**Affiliations:** ^1^Children’s Hospital Colorado, University of Colorado School of Medicine, Aurora, CO, USA

**Keywords:** Cardiac resynchronization therapy, congenital heart disease, hypertrophic cardiomyopathy, leadless pacing, sudden cardiac death

## Introduction

Over the past year, new guidelines for device therapy as well as advances in physiologic pacing, leadless pacing, and risk prediction of sudden cardiac death (SCD) in hypertrophic cardiomyopathy (HCM) in pediatric patients have arisen. Here, we review some the most relevant studies of arrhythmias and pacing in pediatric patients and those with congenital heart disease (CHD).

## New recommendations

The Pediatric and Congenital Electrophysiology Society consensus statement on the indications and management of cardiovascular implantable electronic devices (CIEDs) in pediatric patients^1^ offered several new and interesting recommendations. For catecholaminergic polymorphic ventricular tachycardia, pharmacologic therapy and/or cardiac sympathetic denervation without an implantable cardioverter-defibrillator (ICD) may be considered an alternative in patients presenting with sudden cardiac arrest (SCA). This was made to acknowledge the significant rate of inappropriate device discharge (20%–30%) in this patient population with an apparent lack of survival benefit, even in those presenting with SCA.^2^

Primary-prevention ICD placement was also explored, with several subsections noting that there were inadequate published data to support ICD implantation in pediatric patients with non-ischemic dilated cardiomyopathy (NIDCM) and a left ventricular (LV) ejection fraction (EF) of <35% in the absence of other risk factors. Additionally, patients with NIDCM and impaired LVEF do not benefit from cardiac resynchronization therapy (CRT) in the setting of a narrow QRS.

In patients with indications for implantation of a CIED, shared decision-making and patient-/family-centered care are endorsed and emphasized. Treatment decisions are based on the best available evidence and patient’s preferences.

## Cardiac resynchronization and physiologic pacing

CRT is an established and well-documented therapeutic intervention in adult patients with decreased LVEF and either chronic ventricular pacing or prolonged QRS durations. Conduction system pacing (CSP) has emerged as a sought-after strategy in patients requiring permanent cardiac pacing, and whether it is equivalent, superior, or inferior to biventricular pacing remains unclear. The effect for pediatric patients or those with CHD is even less well defined. Challenges remain as demonstrated by Cano et al.^3^ in a study of 20 patients with CHD in whom CSP was attempted. Only 75% of patients were able to successfully achieve CSP, with the majority (n = 10) receiving His-bundle pacing systems. It is likely that certain anatomic substrates will be more amenable, and patients with congenitally corrected transposition seem particularly well suited to His-bundle pacing.^4^

The long-term outcomes of CRT in CHD remain unclear, though published data are slowly emerging. In a study of 30 patients, Kubuš et al.^5^ reported a modest overall probability of an uneventful CRT continuation (median, 8.7 years) reflecting the complexity of device therapy in this diverse population. There was a significant increase in EF/fractional area of change (*P* < .001) mainly attributable to patients with systemic left ventricle (*P* = .002) and a reduction in systemic ventricular end-diastolic dimensions (*P* < .05) after CRT.

Although patients with systemic right ventricle (SRV) represent a significant proportion of patients with CHD implanted with CRT devices, there are very limited and conflicting data in this specific patient group. Favoring CRT in this population, Jacquemart et al.^6^ assessed CRT patients from 6 French centers, including 31 patients with SRV. The proportions of CRT responders at 6, 12, and 24 months were 82.6%, 80.0%, and 77.8% in patients with SRV versus 66.7%, 64.3%, and 69.6% in patients with systemic left ventricle, respectively (*P* = not significant). The reduction in QRS duration and number of complications were also comparable between the two groups.

## Leadless pacemakers

Leadless transcatheter pacing (LTP) systems have changed the landscape for the adult pacemaker population. Despite the notable benefits of LTP systems, their adoption remains limited in the pediatric population secondary to a large delivery system, potential difficulty in device targeting in a small heart, and uncertainty surrounding late removal. In addition, the only device with atrioventricular synchronous capability (Micra AV; Medtronic, Minneapolis, MN, USA) has limited effectiveness at higher heart rates, which are often physiologic in pediatric patients.

**Figure 1:**
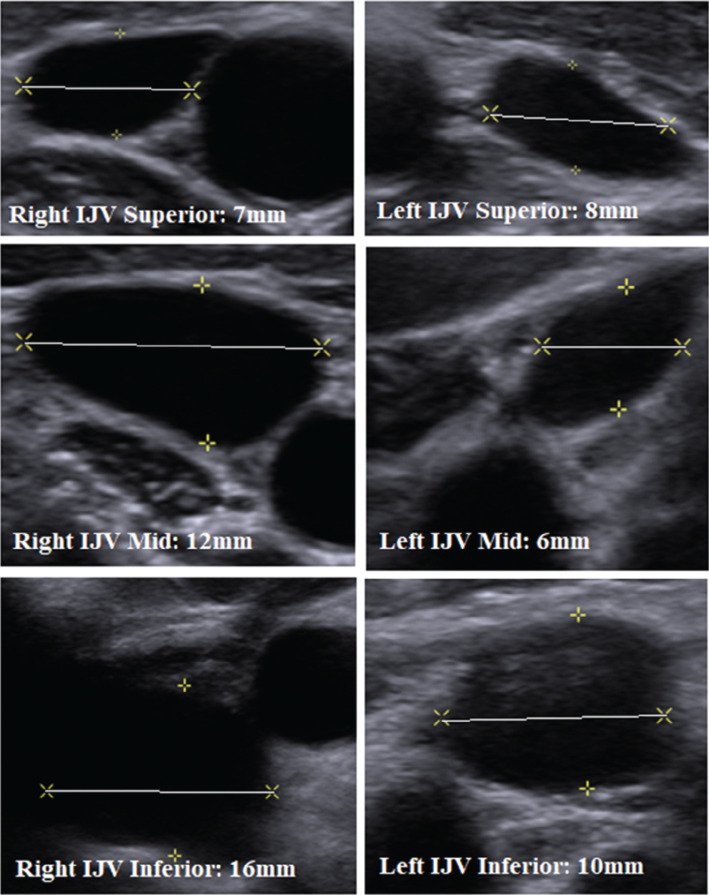
To accommodate a 27-French delivery system, the minimum vessel diameter required is 9 mm. As demonstrated, the right internal jugular vein could comfortably accommodate the delivery sheath in the inferior portions of the vessel and was thus chosen as the route of delivery for the Micra™ transcatheter pacing system. *Abbreviation:* IJV: internal jugular vein. Reproduced with permission from Hackett G, Aziz F, Samii S, Imundo JR. Delivery of a leadless transcatheter pacing system as first-line therapy in a 28-kg pediatric patient through proximal right internal jugular surgical cutdown. *J Innov Card Rhythm Manag*. 2021;12(4):4482–4486.

Despite these drawbacks, LTP systems have been used in carefully selected pediatric patients. Hackett et al. described the implantation of a Micra™ VR LTP system in a 28-kg, 9-year-old patient by surgical cutdown of the right internal jugular vein.^7^ This hybrid approach, with their systematic approach to vessel assessment with preprocedural ultrasound imaging nicely described in the article, is contrasted with the femoral vein approach, which has been used in patients as small as 16 kg.^8^ It has been suggested that a minimum vessel diameter of 9 mm is required for the 27-French (Fr) delivery sheath, though the distensibility of the femoral vasculature has been demonstrated by proponents of the traditional femoral approach in even the smallest patients **(Figure 1)**.

Whether LTP systems represent a viable long-term solution for pediatric patients remains to be seen. Most limiting is that only ventricular pacing is currently available, which severely restricts the indications available for pediatric patients. In their current state, LTP systems appear most suitable for those who infrequently require ventricular pacing, have permanent atrial fibrillation with bradycardia, or have tachycardia–bradycardia syndrome. The addition of a VDD algorithm to the Micra™ platform was encouraging, but its utility at heart rates above 115 bpm is questionable,^9^ and there are currently no available options for leadless pacing in the atrium.

Although a leadless pacemaker is theoretically retrievable, only limited experience has been reported with retrieval after chronic implantation, and these devices can become encapsulated in cardiac tissue.^10,11^ The need for several potential lead extractions over the lifetime of a transvenous system implanted in childhood is frequently cited as a major limitation to eager adoption, and, in their current state, leadless pacemakers do not effectively solve this problem.

## Hypertrophic cardiomyopathy

SCD is the most common mode of death in pediatric HCM, and studies have shown ICDs to be effective. Current guidelines recommend ICD implantation in children with HCM based upon the presence of risk factors for malignant ventricular arrhythmias. A validated risk-prediction model for the prediction of SCD in HCM was presented^12^ by Miron et al. with age at diagnosis, documented non-sustained ventricular tachycardia, unexplained syncope, septal diameter *z*-score, LV posterior wall diameter *z*-score, left atrial diameter *z*-score, peak LV outflow tract gradient, and the presence of a pathogenic variant all being risk factors. Unlike in adults, LV outflow tract gradient had an inverse association and family history of SCD had no association with SCD, respectively.

Notably, patients included in this cohort had a high 5-year cumulative SCD risk of 9.1%. The authors acknowledge this finding by emphasizing that their study focused on an early-onset pediatric group, which would be expected to have a different event rate than that including older patients. Importantly, genotype-positive subjects who did not have echocardiographic evidence of LV hypertrophy and patients with known or suspected secondary causes of HCM—such as clinical syndromes like RASopathies; endocrine, metabolic, mitochondrial, or neuromuscular disorders; hypertension; and structural heart defects—were excluded.

Despite potential improvements in identifying patients who may benefit from ICD implantation, optimal device choice, programming strategies, and appropriate patient selection remain challenging. For the cohort, only 25% received an ICD that was able to abort the event. Additionally, of the 102 patients in the cohort who received a primary-prevention ICD, 86% did not receive an appropriate shock, including many with 5 years of follow-up. This is comparable to adult studies and highlights a major gap in the knowledge about how to risk-stratify for sudden death in the pediatric population.
